# Efficacy and safety of orforglipron, an oral small-molecule GLP-1 receptor agonist, on cardiometabolic outcomes: a meta-analysis and systematic review

**DOI:** 10.1186/s40842-025-00270-4

**Published:** 2026-02-20

**Authors:** Adir Alper, Gal Peleg, Adina Fagin, Priyansh Shah, Ishmum Chowdhury, Antony Gonzales, Robert Faillace

**Affiliations:** 1 Jacobi Medical Center, AECOM, 4857 Broadway, New York, NY 10034 USA; 2 MedStar Washington Hospital Center, Washington, DC USA

**Keywords:** Orforglipron, Oral GLP-1 receptor agonist, Type 2 diabetes mellitus, Cardiometabolic risk

## Abstract

**Background:**

Obesity and type 2 diabetes are the primary drivers of atherosclerotic cardiovascular disease (ASCVD), the leading cause of death worldwide. Injectable GLP-1 receptor agonists reduce major adverse cardiovascular events. Yet for many individuals, injection hesitancy remains a significant barrier to long-term adherence. Orforglipron is a novel once-daily oral non-peptide GLP-1 receptor agonist designed to provide comprehensive cardiometabolic risk reduction.

**Methods:**

PRISMA-compliant systematic review and meta-analysis (PROSPERO CRD420251229397) of placebo-controlled phase 2 and phase 3 trials of orforglipron. Random-effects models were used to pool mean differences (MD) and risk ratios (RR) with 95% confidence intervals.

**Results:**

Across four large trials, orforglipron produced dose-dependent, clinically meaningful improvements in major modifiable cardiovascular risk factor compared with placebo: body weight − 6.08% (95% CI − 7.68 to − 4.47; up to − 9.31% at higher doses), HbA1c − 0.85% (95% CI − 1.53 to − 0.18; up to − 1.36%), systolic blood pressure − 4.32 mmHg (95% CI − 5.61 to − 3.03; up to − 5.78 mmHg at 45 mg), LDL-cholesterol − 4.14% (95% CI − 6.38 to − 1.91), triglycerides − 10.90% (95% CI − 14.36 to − 7.43), VLDL-cholesterol − 10.81% (95% CI − 14.10 to − 7.51), and HDL-cholesterol + 3.31% (95% CI + 1.66 to + 4.97). Notably, heterogeneity was very low to absent (I² = 0%) for systolic blood pressure and all lipid outcomes. Gastrointestinal side effects were common but typical of the GLP-1 class (nausea RR 5.22, vomiting RR 3.24, eructation RR 6.80).

**Conclusion:**

Orforglipron provides highly consistent reductions across the full spectrum of ASCVD risk factors, with effect sizes on lipids and blood pressure comparable to those linked to MACE reduction in trials of injectable GLP-1 receptor agonists. As a novel small-molecule GLP-1 agonist in its class, Orforglipron offers a transformative option for comprehensive cardiometabolic risk reduction and ASCVD prevention in patients with obesity and type 2 diabetes.

**Supplementary Information:**

The online version contains supplementary material available at 10.1186/s40842-025-00270-4.

## Introduction

The twenty-first century is experiencing a pandemic of intertwined cardiometabolic disorders, principally Type 2 Diabetes Mellitus (T2D) and obesity [1, 2], driving atherosclerotic cardiovascular disease (ASCVD), the leading cause of global mortality [3, 4]. Shared pathophysiology, including insulin resistance, chronic inflammation, and endothelial dysfunction, requires therapies targeting comprehensive risk reduction [5, 6]. Incretin-based therapies that leverage gut-derived glucagon-like peptide-1 (GLP-1) have transformed this paradigm [6, 7].GLP-1 Receptor Agonists (GLP-1 RAs), injectable peptide analogues resistant to Dipeptidyl Peptidase-4 (DPP-4) degradation [8] have demonstrated cardiovascular protection in recent cardiovascular outcome trials: LEADER (liraglutide) [9], SUSTAIN-6 (Semaglutide) [10], and REWIND (Dulaglutide) [11], reducing MACE by 12–15% via risk factor improvement and pleiotropic vascular effects [12]. Despite injectable GLP-1 receptor agonists’ proven efficacy and cardioprotection, needle anxiety limits adherence. Oral agents like orforglipron overcome this barrier, potentially improving persistence by 20–30% and broadening ASCVD prevention [13, 14]. Oral semaglutide, co-formulated with Sodium N-(8-[2-hydroxylbenzoyl] amino) caprylate (SNAC), achieved marked bioavailability but requires fasting and 30-minute post-dose restrictions, reducing flexibility [13, 15]. Small-molecule, non-peptide GLP-1 RAs eliminate peptide limitations, enabling oral administration, improving stability, reducing manufacturing costs, and potentially enhancing patient adherence [16–18]. Orforglipron (LY3502970), a potent full agonist with G protein-biased signaling, supports once-daily oral dosing. Preclinical studies showed cAMP stimulation, glucose-lowering, and appetite suppression comparable to exenatide [19, 20], Phase 1a confirmed tolerability and pharmacodynamics in healthy subjects, including weight loss and reduced glucose excursion [21]. Building on this, the Phase 1b, multiple-ascending-dose study in patients with T2D further confirmed dose-dependent improvements in glycemic control and body weight reduction over 12 weeks, providing the first critical evidence in the target population [22]. Phase 2 in T2D achieved up to 1.6% HbA1c and 10.1% weight reduction at 26 weeks [23]. ACHIEVE-1 (Phase 3) in early T2D confirmed significant HbA1c reduction and weight loss at 40 weeks, solidifying its role as a reliable treatment option for glycemic control and weight management [24].

After efficacy was proven in patients with T2D, the utility of Orforglipron in dedicated weight management of patients without T2D was rapidly explored. In non-diabetic obesity, Phase 2 yielded > 10% weight loss at 26 weeks [25]. This magnitude of weight loss compares favorably with established injectable GLP-1 RAs and dual agonists [26]. Furthermore, the confirmatory Phase 3 ATTAIN-1 trial in the non-diabetic obesity population demonstrated sustained, clinically transformative weight reduction over 72 weeks. This trial reinforced the high efficacy and consistent dose-responsive relationship of Orforglipron as a standalone weight management therapy [27]. Importantly, across both the T2D and obesity development programs, exploratory analyses confirmed that Orforglipron is associated with favorable changes in multiple secondary cardiovascular risk biomarkers, including improvements in blood pressure, lipid profiles, and systemic inflammatory markers, suggesting the potential for broad cardiometabolic benefit beyond weight or glucose reduction [28].

Although individual Phase 2/3 trials and previous meta-analyses have established the efficacy of Orforglipron on glycemic control and weight loss in patients with type 2 diabetes and/or obesity, the evidence base remains fragmented across multiple reports, diverse dosing regimens (ranging from 3 mg to 45 mg), and varied treatment durations. A synthesized, quantitative appraisal of the totality of the clinical trial evidence is essential for two reasons. To powerfully estimate unified treatment effects on HbA1c, cholesterol, blood pressure, and weight loss independent of dose, while extending the insights gained from Orfoglipron by systematically evaluating its impact on blood pressure reduction and ASCVD risk stratification ultimately offering clinicians, quantitative evidence to guide cardio metabolic and cardiovascular risk management [29].

## Methods

### Protocol and reporting guidelines

This systematic review and meta-analysis followed PRISMA 2020 guidelines [30]. The protocol was prospectively registered in the International Prospective Register of Systematic Reviews (PROSPERO; registration number: CRD420251229397).

### Inclusion criteria and outcomes

#### Population

Adults ≥ 18 years with type 2 diabetes (T2D) or overweight/obesity (BMI ≥ 27 kg/m² with ≥ 1 weight-related comorbidity or BMI ≥ 30 kg/m²).

#### Intervention

Any dose of orforglipron (LY3502970).

#### Comparator

Placebo.

Study Design: Phase 2 or 3 randomized controlled trials (full-text publications only). Exclusions: Phase 1 trials, observational studies, non-randomized trials, post-hoc analysis and conference abstracts without full-text publication. Required Outcomes: At least one of the primary or key secondary outcomes listed below.

### Primary outcomes

1. Percent change in body weight and waist circumference from baseline to end of treatment.
2. Change in HbA1c (%) from baseline to end of treatment.

### Key secondary outcomes

3. Changes in cardiovascular risk markers: systolic blood pressure (mmHg), diastolic blood pressure (mmHg), total cholesterol, LDL-C (mg/dL), HDL-C (mg/dL), VLDL-C (mg/dL), and triglycerides (mg/dL).
4. Incidence of gastrointestinal adverse events: nausea, vomiting, constipation, diarrhea, headache, dyspepsia, abdominal pain, GERD, and eructation (using standardized terminology of similar MedDRA terms) to ensure uniformity.

### Search strategy and data sources

Databases: MEDLINE/PubMed, Embase, CENTRAL, Web of Science. Supplemented by ClinicalTrials.gov and reference lists. Search up to October 2025; no language restrictions. Strategy: The final search strategy utilized a combination of controlled vocabulary (MeSH and Emtree terms) and text words: (Orforglipron OR LY3502970) AND (clinical trial[Publication Type] OR random*[Title/Abstract] OR placebo[Title/Abstract] OR trial[Title/Abstract] OR study[Title/Abstract])

### Study selection and data extraction

Duplicates removed; two reviewers (FA, PG) independently screened titles/abstracts and full texts. Disagreements resolved by consensus or (AA). Characteristics of includes studies can be seen in (Table S1**)**. PRISMA flow diagram used. Standardized form extracted study characteristics, demographics, interventions, and outcomes (mean change, SD, events). The process will be visually represented using a PRISMA flow diagram (Figure S1) [31].

### Risk of bias and certainty assessment

The methodological quality of all included RCTs was independently assessed by R1 and R2 using the Cochrane Risk of Bias Tool, version 2.0 (RoB 2.0) [32]. Each study received an overall rating of ‘Low risk,’ ‘High risk,’ or unknown risk of bias across five domains (Figure S2).

The certainty of the evidence for the pooled estimate of each primary and key secondary outcome was assessed using the Grading of Recommendations Assessment, Development and Evaluation (GRADE) approach (Table S2) [33].

### Data synthesis and statistical analysis

#### Statistical methods

The meta-analysis employed a random-effects model (DerSimonian-Laird method) to pool effect estimates, accounting for clinical heterogeneity. Dichotomous outcomes were pooled using risk ratios (RR) with 95% confidence intervals (CIs), while continuous outcomes used mean differences (MD) or standardized mean differences (SMD) with 95% CIs. All trials reported mean changes with SDs or SEs (converted to SDs as SD = SE × √n), except HbA1c in Wharton 2023 (missing SD; no formal imputation to avoid assumptions).

Pre-specified subgroup analyses by orforglipron dosage explored dose-response relationships, effect consistency, and heterogeneity sources across the dose range (3–45 mg). Doses were categorized by final maintenance levels (3 mg, 12 mg, 24 mg, 36 mg, 45 mg, where available). Subgroups with data from fewer than two trials per outcome were not pooled to prevent unstable estimates.

### Heterogeneity

Statistical heterogeneity was assessed using the I² statistic (I² > 50% indicating substantial heterogeneity) and chi-square (χ²) test (*p* < 0.10). Pre-specified subgroup analyses explored heterogeneity by orforglipron dosage. Leave-one-out sensitivity analyses were conducted for outcomes with substantial heterogeneity (I² > 50%), but only when ≥ 3 studies were included; otherwise, they were omitted due to limited informativeness, low power, risks of overinterpretation or spurious findings, and low utility, per Cochrane Handbook and common practice in small meta-analyses. Funnel plots were not performed due to the small number of studies (*n* < 10) [34].

### Software

Analyses will be performed using Review Manager (RevMan 5.4) (The Cochrane Collaboration, 2020).

## Results

This meta-analysis consists of four trials in obesity or T2D patients shows that oral Orforglipron, a non-peptide GLP-1RA, delivers consistent, clinically meaningful weight loss and glycemic improvements [23–25, 27].

### Effect of orforglipron on body weight reduction

**Fig. 1:**
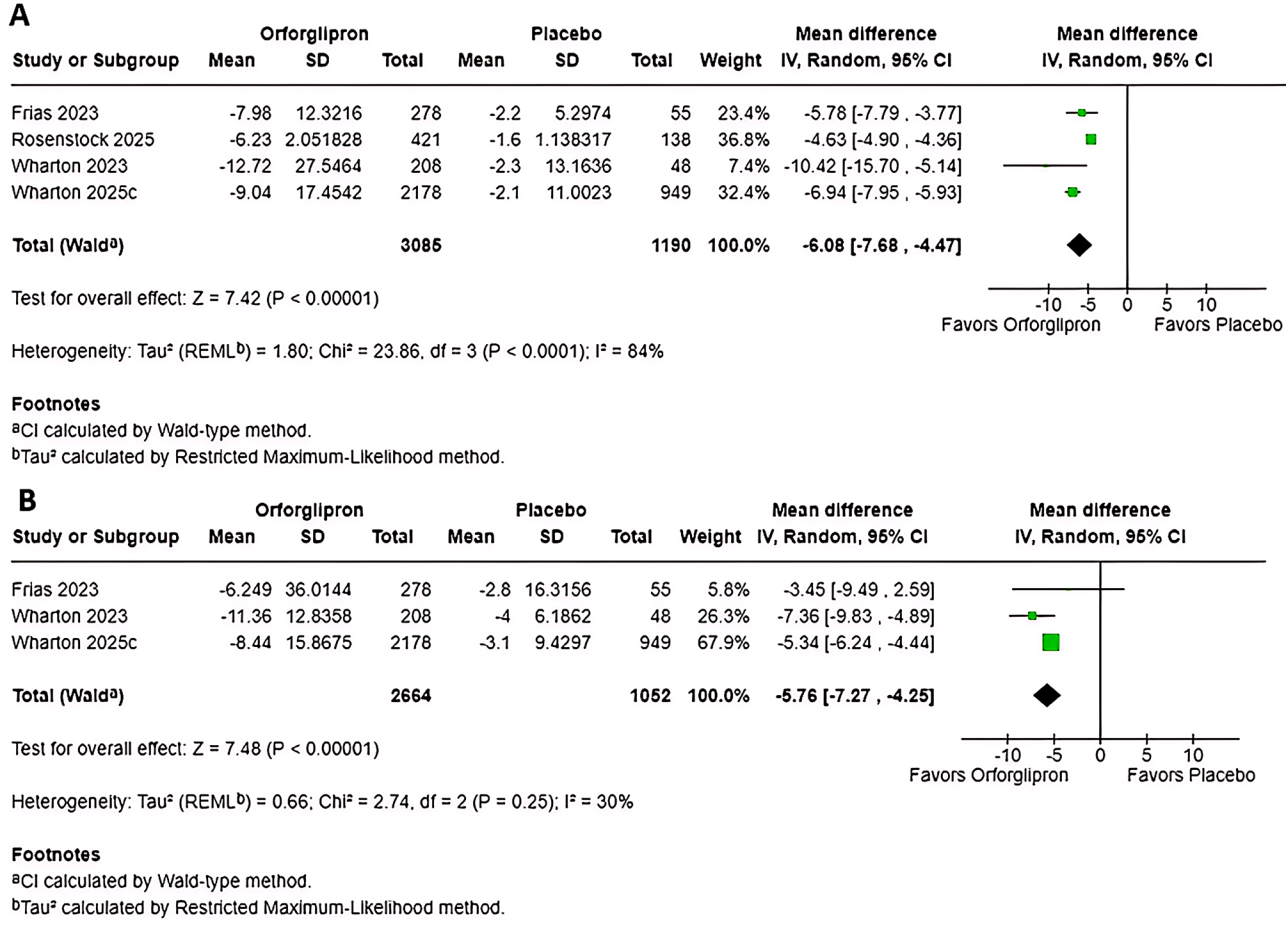
Effect of orforglipron on body weight and waist circumference. Forest plots showing the pooled mean difference in (**A**) percentage change in body weight from baseline and (**B**) change in waist circumference (cm) from baseline with orforglipron versus placebo. Analyses were performed using a random-effects model. Squares represent mean differences for individual studies, with size proportional to study weight; horizontal lines represent 95% confidence intervals. Diamonds represent pooled mean differences and 95% CIs overall

The pooled meta-analysis of four studies demonstrated a highly significant reduction in body weight with Orforglipron compared to placebo (MD: -6.08%; 95% CI, -7.68 to -4.47; *P* < 0.01) (Fig. 1). However, the overall estimate exhibited substantial heterogeneity (*I*² = 84%; *P* < 0.01), indicating variability across trials. Excluding Rosenstock et al. (2025) reduced heterogeneity to I² = 0%, with the pooled estimate shifting to MD -6.81 (95% CI -7.70 to -5.92), which maintains the statistical significance of the result. To explore this, a subgroup analysis was performed (Figure S3.). This analysis confirmed a strong, dose-dependent relationship (*P* < 0.01, *I*² = 87.6%), with all doses achieving significant weight loss. The clinically relevant threshold of > 5% weight loss was first reached at the 12 mg dose (MD: -5.20%). The higher doses (24 mg, 36 mg, and 45 mg) resulted in mean weight reductions exceeding − 7.8%. Notably, the 24 mg dose was the only subgroup to demonstrate negligible heterogeneity (*I*² = 0%), providing the most consistent effect estimate. Owing to the small number of eligible studies, sensitivity analyses were not performed.

**Fig. 2:**
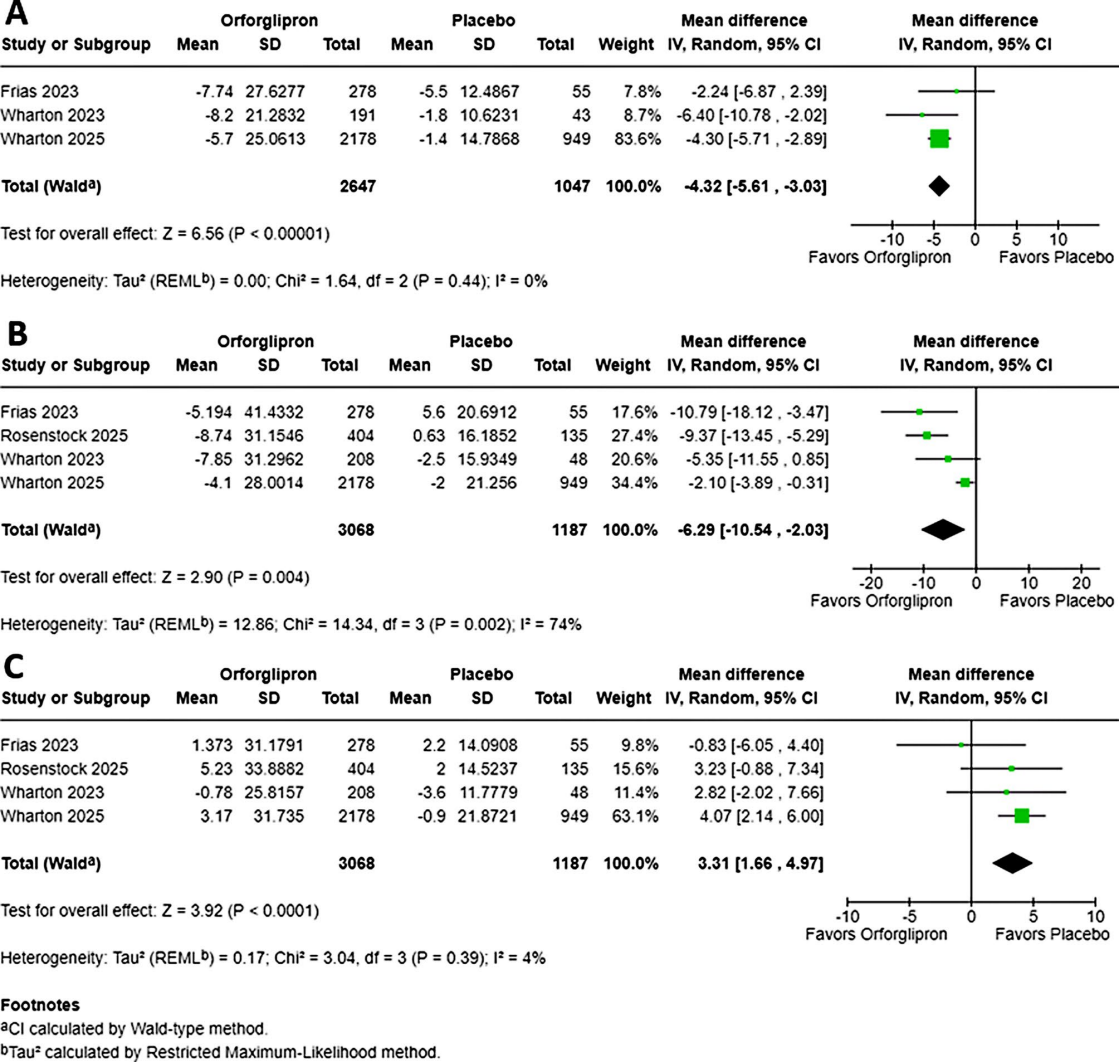
Effect of orforglipron on systolic blood pressure, total cholesterol, and HDL cholesterol. Forest plots showing the pooled mean difference in (**A**) change in systolic blood pressure (mmHg), (**B**) percentage change in total cholesterol, and (**C**) percentage change in HDL cholesterol from baseline with orforglipron versus placebo. Analyses were performed using a random-effects model. Squares represent mean differences for individual studies, with size proportional to study weight; horizontal lines represent 95% confidence intervals. Diamonds represent pooled mean differences and 95% CIs overall

**Fig. 3:**
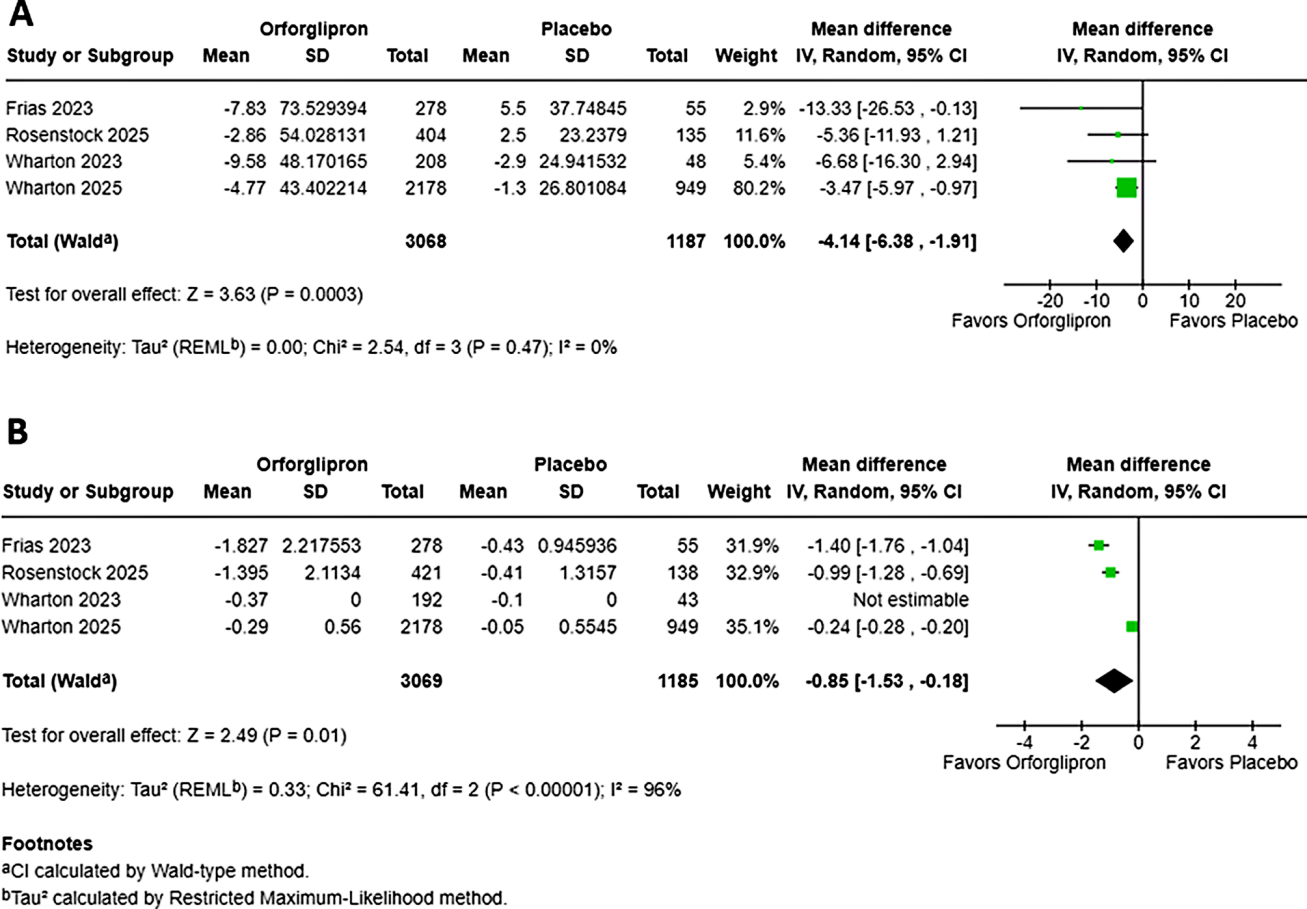
Effect of orforglipron on LDL Cholesterol and HbA1C. Forest plots showing the pooled mean difference in percentage change from baseline in (**A**) LDL cholesterol, (**B**) HbA1C with orforglipron versus placebo. Analyses were performed using a random-effects model. Squares represent mean differences for individual studies, with size proportional to study weight; horizontal lines represent 95% confidence intervals. Diamonds represent pooled mean differences and 95% CIs

**Fig. 4:**
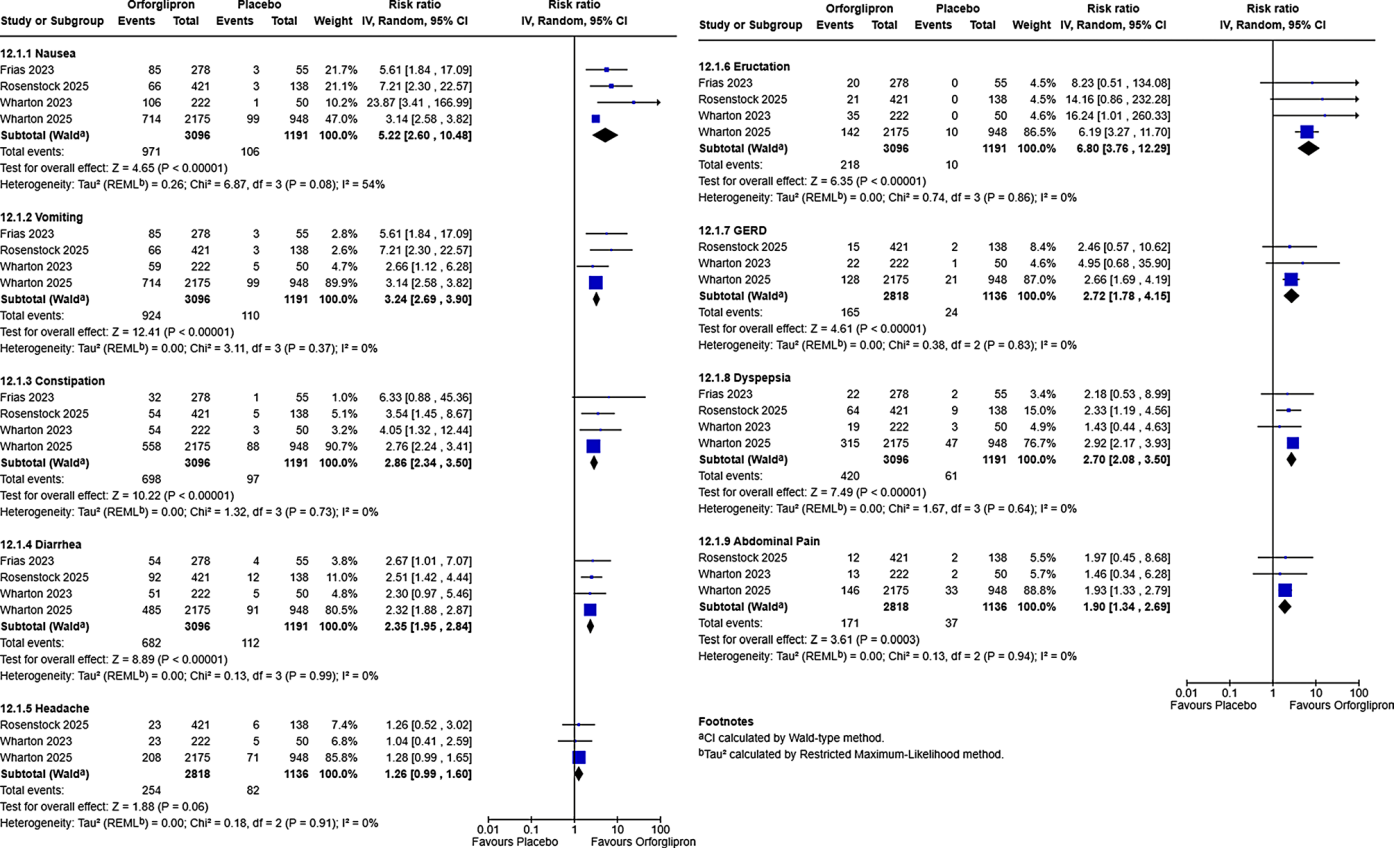
Gastrointestinal safety profile of orforglipron versus placebo. Forest plot of risk ratios for common gastrointestinal adverse events with orforglipron versus placebo. Analyses were performed using a random-effect. Squares represent risk ratios for individual studies, with size proportional to study weight; horizontal lines represent 95% confidence intervals. Diamonds represent pooled risk ratios and 95% CIs

### Effect of orforglipron on waist circumference

A pooled analysis of three studies demonstrated a statistically significant reduction in waist circumference (WC) with Orforglipron (MD: -5.76 cm; 95% CI, -7.27 to -4.25; *P* < 0. 01) (Fig. 1). The estimate was consistent and showed low between-study heterogeneity across trials (*I*² = 30%; *P* = 0.25). A subgroup analysis (12 mg to 45 mg) confirmed consistency in effect magnitude across higher doses (*P* = 0.50, *I*² = 0%), with no significant difference between subgroups (Figure S4). The greatest mean reductions in waist circumference (–6.11 cm to − 6.24 cm) were observed in the 24 mg, 36 mg, and 45 mg groups, suggesting that maximal efficacy on central adiposity plateaus at or around the 24 mg dose, which also yielded the most precise estimate (I² = 0%). Subgroup sensitivity analysis was not performed due to the small number of studies.

### Effect of orforglipron on systolic blood pressure (SBP)

The pooled analysis of all Orforglipron doses demonstrated a statistically and clinically significant reduction in SBP compared to placebo. The overall pooled Mean Difference (MD) in SBP reduction was − 4.32 mmHg (95% CI, -5.61 to -3.03; *P* < 0.01) (Fig. 2). The magnitude of SBP reduction was substantial, and its consistency across subgroups was confirmed by the low heterogeneity across the included studies (*I*² = 0%). The observed I² = 0% should be interpreted cautiously because, with only 3–4 trials, the statistical power to detect heterogeneity is low and confidence intervals around I² are wide.

A subgroup analysis across four major dosing regimens (12 mg, 24 mg, 36 mg, and 45 mg) revealed a generally dose-dependent trend (Figure S5). All dose groups showed a significant SBP reduction, with the highest dose (45 mg) yielding the greatest effect (MD: -5.78 mmHg). Importantly, most individual dose subgroups (12 mg, 24 mg) demonstrated negligible heterogeneity (*I*² = 0%), reinforcing the highly consistent SBP-lowering effect of Orforglipron at various stable doses.

### Effect of orforglipron on diastolic blood pressure (mmHg)

The pooled analysis across three studies showed a statistically significant reduction in diastolic blood pressure (DBP) (MD: -1.00 mmHg; 95% CI, -1.03 to -0.97; *P* < 0.01) (Figure S6). While the effect size was small, consistency was high, with low and non-significant heterogeneity across studies (*I*² = 0%; *P* = 0.54). This suggests Orforglipron may not have a clinically meaningful overall impact on DBP across the dose range studied (Figure S7). The subgroup analysis across four dose levels (12 mg to 45 mg) indicated that the effect on DBP did not vary significantly between doses (Test for subgroup differences: *P* = 0.42, *I*² =0%). Only the 36 mg dose achieved a statistically significant DBP reduction (MD: -1.13 mmHg; 95% CI, -1.98 to − 0.28; *P* = 0.01).

### Effect of orforglipron on total cholesterol (%)

The pooled meta-analysis of four studies demonstrated a statistically significant reduction in total cholesterol with Orforglipron (MD: -6.29%; 95% CI, -10.54 to -2.03; *P* < 0.01) (Fig. 2). This overall effect, however, was accompanied by substantial statistical heterogeneity across trials (*I*² = 74%; *P* = 0.002). When the study by Wharton 2025 was excluded, heterogeneity dropped markedly to I² = 0%, and the pooled effect changed to MD -8.62% (95% CI -11.72 to -5.53). The influence analysis, therefore, identified Wharton 2025 as an outlier, potentially due to differences in the trial population (e.g., non-diabetic obesity focus). A subsequent subgroup analysis across five dosing regimens (3 mg to 45 mg) indicated that the magnitude of the cholesterol-lowering effect did not significantly differ between dose groups (*P* = 0.85, *I*² = 0%), suggesting efficacy on total cholesterol is not strictly dose-dependent (Figure S8).

### Effect of orforglipron on HDL cholesterol

The pooled meta-analysis of four studies demonstrated a statistically significant increase in HDL cholesterol with Orforglipron (MD: +3.31%; 95% CI, 1.66 to 4.97; *P* < 0.01) (Fig. 2). This positive effect was characterized by low heterogeneity across trials (*I*² = 4%; *P* = 0.39). The subgroup analysis across five dosing regimens (3 mg to 45 mg) indicated that the HDL-increasing effect did not vary significantly by dose (*P* = 0.59, *I*² = 0%). Only the 12 mg (MD: 3.34; *I*² = 0%) and 36 mg (MD: +3.67%; *I*² = 32%) doses showed a statistically significant increase in HDL cholesterol (Figure S9). Conversely, the 24 mg and 45 mg doses were non-significant, suggesting that the positive lipid effect is not strictly dose-dependent and may reach maximal impact regardless of dosage. In the 3 mg subgroup (I² = 78%), due to the limited number of included studies (*n* = 2), a leave-one-out sensitivity analysis could not be performed.

### Effect of orforglipron on LDL cholesterol

The pooled meta-analysis of four studies demonstrated a statistically significant reduction in LDL cholesterol with Orforglipron (MD: -4.14%; 95% CI, -6.38 to -1.91; *P* < 0.01) (Fig. 3). This LDL-lowering effect was highly reliable, evidenced by the low and non-significant heterogeneity across trials (*I*² = 0%; *P* = 0.47). A subgroup analysis across five dosing regimens (3 mg to 45 mg) suggested that the magnitude of the LDL-lowering effect did not statistically differ by dose (*P* = 0.44, *I*² = 0%) (Figure S10). However, all doses above 3 mg demonstrated a statistical significant reduction. Notably, the 24 mg dose showed the largest decrease (MD: -10.40%) with zero statistical heterogeneity (*I*² = 0%), confirming a highly consistent LDL-lowering effect at this dosage. A notable mean reduction was observed at the 3 mg dose (MD: -10.39%), although this was associated with substantial heterogeneity (*I*² = 72%). Leave-one-out sensitivity analysis was not feasible owing to the small number of eligible studies (k = 2).

### Effect of orforglipron VLDL cholesterol (%)

The pooled meta-analysis demonstrated a highly significant reduction in VLDL cholesterol with Orforglipron (MD: -10.81%; 95% CI, -14.10 to -7.51; *P* < 0.01) (Figure S11). Crucially, the analysis showed zero statistical heterogeneity across all included studies (*I*² = 0%; *P* = 0.92), supporting good consistency of these pooled estimates, albeit within the constraints of a meta-analysis comprising only four trials. A subgroup analysis across five dosing regimens (3 mg to 45 mg) suggested a trend toward differential effects across doses (*P* = 0.44, *I*² = 0%) (Figure S12), Although a numerical trend toward greater VLDL cholesterol reduction with higher doses was observed, the absence of statistical significance suggests that a clear dose-response relationship could not be established within the range of doses evaluated.

### Effect of orforglipron on triglycerides

The pooled meta-analysis demonstrated a highly significant reduction in triglycerides (TGs) with Orforglipron (MD: -10.90%; 95%CI, -14.36 to -7.43; *P* < 0.01) (Figure S13). Importantly, the analysis showed zero statistical heterogeneity across all included studies (*I*² = 0%; *P* = 0.93), providing maximal confidence in the consistency of this pooled TG-lowering effect The subgroup analysis across five dosing regimens (3 mg to 45 mg) indicated a significant variation in effect magnitude between doses (*P* = 0.07, *I*² = 54.1%) (Figure S14). A clear dose-dependent trend was apparent, with the maximum effect at the 36 mg dose (MD: -15.58%, *I*² = 0). While the 3 mg dose was non-significant, all doses ≥ 12 mg demonstrated a highly significant TG-lowering effect. Notably, the 3 mg, 12 mg, 24 mg, and 36 mg subgroups all showed zero statistical heterogeneity (*I*² = 0%), establishing these dose-specific effects as highly consistent.

### Effect of orforglipron on HbA1c

The pooled meta-analysis of four studies demonstrated a statistically significant reduction in HbA1c with Orforglipron (MD: -0.85%; 95% CI, -1.53 to -0.18; *P* < 0.01) (Fig. 3). However, the pooled estimate was characterized by significant heterogeneity across trials (*I*² = 96%; *P* < 0.01), Although high heterogeneity was observed (I² = 96%), robustness was confirmed through leave-one-out sensitivity analyses, where pooled HbA1c reductions ranged from − 0.60% to -1.18% (all *P* < 0.01). Subgroup analysis across five dosing regimens (3 mg to 45 mg) demonstrated a significant dose-dependent reduction in HbA1c (*P* < 0.01, *I*² = 93.9%) (Figure S15). The greatest HbA1c reduction occurred at 24 mg (MD -1.36%, 95% CI -1.74 to -0.98) and 36 mg (MD -1.26, 95% CI -2.51 to -0.01), with smaller effects in lower doses (3-12 mg) and at the highest dose of 45. Notably, Wharton 2023 did not report standard deviations, which may have contributed to its influence on heterogeneity.

#### Safety analysis

As illustrated in Fig. 4, the pooled meta-analysis for adverse events consistently demonstrated a significantly elevated risk for a spectrum of gastrointestinal (GI) adverse events with Orforglipron treatment compared to placebo. All pooled analyses for GI AEs except to nausea exhibited zero statistical heterogeneity (*I*² =0%). The most pronounced adverse event was eructation, which exhibited the highest relative risk (RR = 6.80. Significant increases were also observed for constipation (RR = 2.86), vomiting (RR = 3.24), Nausea (RR = 5.22, I² = 54). Additional statistically significant increases were found for diarrhea (RR = 2.35), gastroesophageal reflux disease (GERD) (RR = 2.72), dyspepsia (RR = 2.70), and abdominal pain (RR = 1.90). In contrast, headache did not demonstrate a statistically significant difference between the Orforglipron and placebo groups (RR = 1.26; *P* = 0.06). Leave-one-out analysis for nausea (I² = 54%) showed that excluding Wharton 2025 reduced I² to 0% and increased the pooled RR from 5.22 (95% CI 2.60–10.48) to 7.67 (95% CI 3.67–16.04). The pooled analysis using random-effects of treatment discontinuation due to any adverse event was significantly higher with Orforglipron than with placebo across the four phase 2 and phase 3 trials (RR 2.99, 95% CI 2.07–4.33; *P* < 0.00001; I² = 0%), with no statistical heterogeneity (I² = 0%) (Figure S16). Overall, orforglipron was associated with a nearly threefold higher likelihood of discontinuation due to side effects compared with placebo.

## Discussion

This systematic review and meta-analysis provides the first quantitative synthesis of the cardiometabolic efficacy and safety of orforglipron, the novel oral non-peptide GLP-1 receptor agonist currently in development. In analysis of pooled data from four large phase 2 and phase 3 placebo-controlled trials involving more than 4,500 participants with type 2 diabetes (T2D) or obesity [23–25, 27], orforglipron produced clinically significant, dose-dependent improvements across virtually all modifiable atherosclerotic cardiovascular disease (ASCVD) risk factors: body weight − 6.08% (95% CI -7.68 to -4.47, reaching − 8.12% to − 9.31% at 24–45 mg doses), HbA1c -0.85% overall (95% CI -1.53 to -0.18; up to -1.36% at higher doses), systolic blood pressure − 4.32 mmHg (95% CI -5.61 to -3.03), LDL-cholesterol − 4.14% (95% CI -6.38 to -1.91), triglycerides − 10.90% (95% CI -14.36 to -7.43), VLDL-cholesterol − 10.81% (95% CI -14.10 to -7.51), and a + 3.31% rise in HDL-cholesterol (95% CI + 1.66 to + 4.97). The very low or absent heterogeneity (I² = 0%) observed for systolic blood pressure, LDL-cholesterol, triglycerides, and VLDL-cholesterol is noteworthy and can suggest a highly predictable class effect driven by orforglipron’s unique non-peptide structure and G-protein-biased signaling [16, 17]. However, with only four trials, confidence intervals around I² are wide, precluding definitive claims of perfect between-study consistency. Heterogeneity in lipid and blood-pressure outcomes (I² up to 74%) was driven by expected population differences: the two T2D trials had higher baseline LDL-C and total cholesterol at baseline and longer duration (72 weeks) than the two non-diabetic obesity trials (52 weeks), accounting for the larger atherogenic-lipid reductions and smaller HDL increases in the T2D cohort (Table S1).

These effect sizes are clinically meaningful when placed in context. A ≥ 5% body weight reduction, as achieved across all doses ≥ 12 mg, is associated with substantial improvements in comorbidities such as hypertension, dyslipidemia, and sleep apnea [35]. Notably, meta-analyses have shown that a 1 mmol/L reduction in LDL-cholesterol corresponds to a highly significant 22% risk reduction in major cardiovascular events [36]. Similarly, the HbA1c reduction exceeds the 0.5% threshold linked to microvascular risk reduction in T2D guidelines [5]. The pooled systolic blood pressure reduction (-4.32 mmHg; 95% CI -5.61 to -3.03, up to -5.78 mmHg at higher doses) approximates the 5 mmHg threshold associated with an approximately 10% relative risk reduction in major cardiovascular events [37]. More critically, applying the pooled changes observed in this analysis to the 2013 ACC/AHA Pooled Cohort Equations (or SCORE2-Diabetes in European cohorts), Orforglipron reduces the estimated 10-year ASCVD risk by 15.3–20.5% relative to placebo (Table S3) [38–40]. This magnitude aligns closely with the 12–26% relative risk reductions in major adverse cardiovascular events (MACE) observed in landmark cardiovascular outcome trials of injectable GLP-1 receptor agonists (LEADER, SUSTAIN-6, REWIND, SELECT) [9–11, 41]. However, definitive evidence of cardiovascular protection requires dedicated outcome trials, which have not yet been initiated for Orforglipron. Mediation analyses from these trials attribute 50–70% of the MACE benefit to similar surrogate improvements in weight, HbA1c, blood pressure, and lipids, with the remainder potentially due to pleiotropic effects such as reduced inflammation (e.g., hsCRP reductions of 26–42% observed in orforglipron’s exploratory analyses) [12, 27]. GLP-1 receptor agonists significantly reduce MACE and all-cause mortality. Liraglutide and dulaglutide show the broadest CV benefit (MACE, CV death, MI, stroke, and heart failure), followed by subcutaneous semaglutide and albiglutide; oral semaglutide and exenatide show favorable trends.

Orforglipron, a novel once-daily oral small-molecule GLP-1RA, has not yet reported MACE outcomes from its ongoing CVOT. It remains highly promising for future CV protection due to its non-peptide structure, high bioavailability, food-independent dosing, and superior weight-loss efficacy comparable to injectable agents [42].

Direct comparison with established GLP-1 receptor highlights Orforglipron’s emerging competitive position (Table S17). At higher doses, orforglipron’s weight loss (− 8.25% to − 9.31%) approaches weekly subcutaneous semaglutide 2.4 mg (-12.4%)[37] and clearly exceeds oral semaglutide 14 mg (-3.4%) [10, 43]. In addition, its systolic blood pressure reduction (− 3.2 to − 5.8 mmHg) and lipid effects (LDL-C -10.4% to -8.45%, triglycerides − 13.60% to 14.45%) equal or surpass those of injectable semaglutide and are substantially larger than those reported for oral semaglutide [10, 43, 44]. Despite lower absolute weight loss, Orforglipron’s lipid and blood-pressure benefits approach the magnitude seen with the dual GIP/GLP-1 receptor agonist tirzepatide [45, 46], suggesting that its non-peptide, G-protein-biased signaling profile may confer disproportionate cardiometabolic effects beyond those explained by weight reduction alone [16–18]. Notably, whereas weight loss and HbA1c reduction remain dose-dependent up to 45 mg, beneficial effects on systolic blood pressure, LDL-cholesterol, triglycerides, and VLDL-cholesterol plateau at 12–24 mg with zero heterogeneity. This suggests that maximal ASCVD risk reduction may be achieved at lower doses than those needed for maximal weight loss, likely due to orforglipron’s G-protein-biased signaling that preferentially enhances hepatic and vascular effects relatively independent of central appetite suppression, This mechanism supported by preclinical data indicate enhanced hepatic GLP-1 receptor activation, potentially driving direct triglyceride-lowering via reduced VLDL secretion, independent of caloric intake [19, 20]. This mechanistic distinction likely explains the low heterogeneity in lipid and blood pressure outcomes (I² = 0% for SBP, LDL-C, VLDL-C, and triglycerides), in contrast to the high, dose-driven heterogeneity observed for body weight (I² = 84%) and HbA1c (I² = 96%). Consequently, near-maximal cardiometabolic protection appears achievable at modest doses (12–24 mg), whereas higher doses (≥ 36 mg) are required primarily for optimal glycemic control and weight loss. This profile supports a personalized dosing strategy: lower doses may suffice for cardiovascular risk reduction, while higher doses can be reserved for patients prioritizing intensive weight management or diabetes control. An intriguing pattern emerged in lipid responses across populations. In patients with type 2 diabetes (Frias 2023; Rosenstock 2025), orforglipron produced markedly greater reductions in LDL-cholesterol (-9.4% to -13.3%) and total cholesterol (-9.4% to -10.8% %) than in non-diabetic obesity (-3.5% to -6.7% and − 2.1 to -5.4%, respectively). Conversely, HDL-cholesterol increased more in the non-diabetic obesity trials (+ 2.8 to + 4.1%) than in T2D (-0.8 to + 3.2%), while triglyceride reductions were similar in both groups (-8.3 to -13.9%). This differential profile suggests that orforglipron particularly improves the most adverse lipid fractions in patients with type 2 diabetes, while favoring an HDL-raising effect in non-diabetic obesity, reinforcing its versatile cardiometabolic benefit across the entire spectrum.

Gastrointestinal adverse events remain the principal tolerability limitation, aligning with the GLP-1 receptor agonist class, albeit toward the higher end of the spectrum (nausea RR 5.22 [95% CI 2.60–10.48], vomiting RR 3.24 [95% CI 2.69–3.90], eructation RR 6.80 [95% CI 3.76–12.29]) [44, 47]. A 2024 network meta-analysis ranks orforglipron highest for nausea risk among oral GLP-1 RAs in non-diabetic patients, potentially due to its rapid absorption profile [44]. Overall discontinuation due to any adverse event was 7.8% with orforglipron versus 2.7% with placebo (RR 2.99, 95% CI 2.07–4.33). Importantly, rates were markedly lower in the two phase 3 trials (4.4% − 7.8%) than in phase 2 studies (10% − 17%), reflecting the benefit of slower dose-escalation schedules now implemented in later-stage development [23–25, 27]. Orforglipron’s non-peptide structure eliminates the need for SNAC and fasting restrictions, delivering absolute oral bioavailability of 20–40% compared with only 0.4–1% for oral semaglutide. This yields markedly more consistent systemic exposure resulting in pharmacokinetic stability that contributes to the near-absent heterogeneity observed in systolic blood pressure and lipid effects (I² = 0% for SBP, LDL-C, triglycerides, and VLDL-C), a clear advantage over current oral peptide formulations [10, 13, 15, 22]. This practical advantage may translate to improved long-term adherence, an outcome repeatedly identified as the major barrier to sustained benefit with current injectable therapies in real-world registries [47].

Beyond efficacy and safety, orforglipron’s profile holds particular promise for broadening access in underserved populations. In the global obesity-T2D pandemic, where ASCVD disproportionately affects ethnic minorities and low-income groups with limited healthcare access [1–4], an affordable oral agent without refrigeration or injection needs could bridge equity gaps. Exploratory data from phase 3 trials show consistent benefits across subgroups (e.g., BMI ≥ 35 kg/m², HbA1c > 8%) [27, 28] and its non-peptide stability may lower manufacturing costs, facilitating scalability in resource-constrained settings [17, 18]. If approved (with FDA submissions anticipated in late 2025), orforglipron could complement lifestyle interventions in primary prevention, especially where there are barriers to subcutaneous injections.

The strengths of this meta-analysis include the incorporation of the most recent phase 3 data (ACHIEVE-1 and ATTAIN-1) [24, 27], detailed dose–response analyses that resolve sources of heterogeneity (e.g., via leave-one-out sensitivity), and the exceptionally consistent effects on blood pressure and lipids (I²=0% for all lipid fractions and SBP), supported by high GRADE certainty ratings. (Table S2). We acknowledge several limitations. Only four trials were available, with the longest extending to 72 weeks, limiting our ability to assess long-term durability or detect rare adverse events. Moreover, hard cardiovascular, renal, and heart-failure outcomes have not yet been reported, and a dedicated CVOT—analogous to SURPASS for tirzepatide—has not been initiated. Surrogate risk equations, while validated, cannot substitute for observed event reduction [39, 40], and the moderate GRADE certainty for weight/HbA1c (due to inconsistency) warrants caution. Finally, with only four studies, funnel plots for publication bias were infeasible.

In conclusion, orforglipron represents a potential paradigm shift in cardiometabolic pharmacotherapy: a once-daily oral small-molecule that combines unparalleled ease of administration with comprehensive, highly consistent reductions in weight, glycemia, blood pressure, and the full spectrum of atherogenic lipids Its effects rival or exceed those of oral semaglutide and approach injectable benchmarks, positioning it as a versatile option for personalized therapy (e.g., 24 mg for weight, 45 mg for SBP). If dedicated cardiovascular outcome trials-expected post-approval will confirm translation of these surrogate improvements into MACE reduction similar to established injectable GLP-1 receptor agonists, orforglipron could dramatically expand access to high-efficacy GLP-1 based therapy, particularly among injection-averse patients and in resource-constrained settings. This novel treatment can favorably modify major drivers of ASCVD risk with a single daily pill positioning it as a transformative candidate for future guideline-directed medical therapy in the escalating global burden of obesity-related cardiovascular disease.

## Supplementary Information

Below is the link to the electronic supplementary material.

Supplementary Material 1

## Data Availability

The datasets generated and/or analyzed during the current study are not publicly available institution policy but are available from the corresponding author on reasonable request.
