# Supplementary material for: Efficacy and safety of orforglipron, an oral small-molecule GLP-1 receptor agonist, on cardiometabolic outcomes: a meta-analysis and systematic review
Source: Cardiovasc Diabetol Endocrinol Rep. 2026 Feb 20;12:9. doi: 10.1186/s40842-025-00270-4 (PMC12922244; doi:10.1186/s40842-025-00270-4)
Supplement: Supplementary file 1 — Supplementary Material 1 [file 40842_2025_270_MOESM1_ESM.docx]

*Systematic Review and Meta-Analysis*

Efficacy and Safety of Orforglipron, an Oral Small-Molecule GLP-1 Receptor Agonist, on Cardiometabolic Outcomes: A Meta-Analysis and Systematic Review

**Supplementary Appendix**

**Figure S1: PRISMA 2020 Flow Diagram**

**
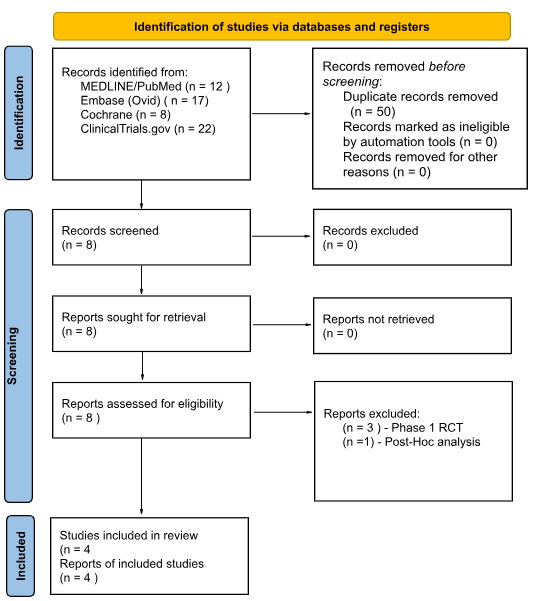
**

Supplemental Figure 1 - Flow diagram of study identification, screening, eligibility assessment, and inclusion according to the PRISMA 2020 statement.

**Figure S2: Risk of Bias Assessment of Included Studies Using Cochrane RoB**

**
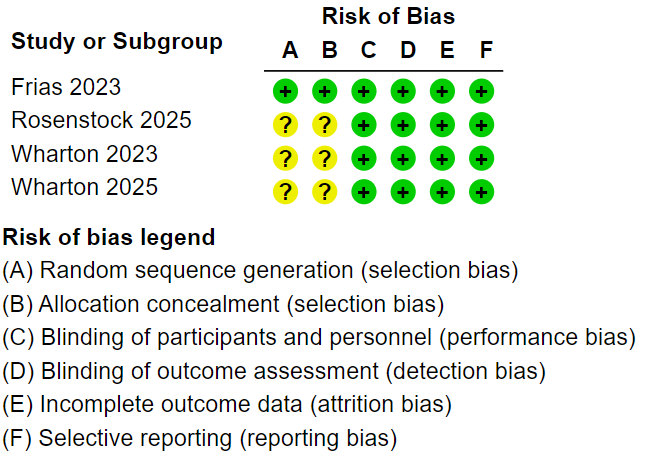
**

Figure S2 - Traffic-light plot (left) and weighted summary plot (right) of risk-of-bias domains (randomization process, deviations from intended interventions, missing outcome data, measurement of the outcome, selection of the reported result) for the our four included randomized controlled trials.

Table S1: Characteristics of Included Studies Evaluating Orforglipron

| Study | Year | Journal | Population | Study Design | Sample size (N, mean per arm) | Intervention | Comparator | Duration | Primary Outcomes | Key Findings |
| --- | --- | --- | --- | --- | --- | --- | --- | --- | --- | --- |
| Frias et al. | 2023 | The Lancet | Adults (≥18 years) with type 2 diabetes treated with diet and exercise ± metformin, HbA1c 7.0–10.5%, BMI ≥23 kg/m² | Multicentre, randomised, double-blind, dose-response, phase 2  NCT05048719 | 383 total, ~54,  9 arms: placebo, dulaglutide, orforglipron | Oral orforglipron 3–45 mg daily | Placebo | 26 weeks | Mean change in HbA1c from baseline to week 26 | Orforglipron doses ≥12 mg reduced HbA1c by up to -2.10% (placebo-adjusted -1.67%) and body weight by up to -10.1 kg (placebo-adjusted -7.9 kg); adverse events mainly gastrointestinal, similar to GLP-1 RAs. |
| Wharton et al. | 2023 | New England Journal of Medicine | Adults (18–75 years) with obesity (BMI ≥30) or overweight (BMI 27–<30) + ≥1 weight-related comorbidity, no diabetes | Randomised, double-blind, placebo-controlled, phase 2  NCT05051579 | 272 total, ~54 per arm (placebo ,12mg, 24mg, 36mg, 34mg, orforglipron) | Orforglipron 12 mg, 24 mg, 36 mg, 45 mg once daily | Placebo | 36 weeks | Percentage change in body weight from baseline to week 26 | Weight reduction -8.6% to -12.6% at week 26 and -9.4% to -14.7% at week 36 with orforglipron vs. -2.3% with placebo; improvements in cardiometabolic measures; gastrointestinal AEs common, leading to 10–17% discontinuation. |
| Rosenstock et al. | 2025 | New England Journal of Medicine | Adults (≥18 years) with early type 2 diabetes inadequately controlled with diet and exercise alone, HbA1c 7.0–9.5%, BMI ≥23 | Multicentre, randomised, double-blind, placebo-controlled, phase 3 (ACHIEVE-1)  NCT05971940 | 700, ~175  (4 arms: placebo, orforglipron 3 mg, 12 mg, 36 mg | Orforglipron 3 mg, 12 mg, 36 mg once daily | Placebo | 40 weeks | Change in HbA1c from baseline to week 40 | HbA1c reductions -1.24% (3 mg), -1.47% (12 mg), -1.48% (36 mg) vs. -0.41% placebo; weight change -4.5% to -7.6% vs. -1.7%; mild-moderate gastrointestinal AEs common; discontinuation 4.4–7.8%. |
| Wharton et al. (2025c) | 2025 | New England Journal of Medicine | Adults (≥18 years) with obesity (BMI ≥30) or overweight (BMI 27–<30) + ≥1 obesity-related complication, no diabetes | Multinational, randomised, double-blind, placebo-controlled, phase 3 (ATTAIN-1)  NCT05803421 | 3700, ~925 (4 arms: placebo, orforglipron 6 mg, 12 mg, 36 mg) | Orforglipron 6 mg, 12 mg, 36 mg once daily | Placebo | 72 weeks | Percentage change in body weight from baseline to week 72 | Weight change -7.5% (6 mg), -8.4% (12 mg), -11.2% (36 mg) vs. -2.1% placebo; improvements in waist circumference, BP, lipids; gastrointestinal AEs mild-moderate, discontinuation 5.3–10.3%. |

**Table S2: Summary of findings and certainty of evidence (GRADE)**

| **Outcome** | **No. of participants (studies)** | **Design** | **Risk of bias** | **Inconsistency** | **Indirectness** | **Imprecision** | **Certainty (GRADE)** | **Pooled Effect (95% CI)** |
| --- | --- | --- | --- | --- | --- | --- | --- | --- |
| **Body weight  (% change)** | 4,275 (4 RCTs) | RCT | Not serious | Serious (–1)¹ | Not serious | Not serious | ⊕⊕⊕◯ Moderate | MD -6.08% (-7.68 to -4.47) |
| **Waist circumference (cm)** | 3,716 (3 RCTs) | RCT | Not serious | Serious (–1)² | Not serious | Not serious | ⊕⊕⊕◯ Moderate | MD -5.76 cm  (-7.27 to -4.25) |
| **HbA1c  (% change)** | 4,254 (4 RCTs) | RCT | Not serious | Serious (–1)³ | Not serious | Not serious | ⊕⊕⊕◯ Moderate | MD -0.85%  (-1.53 to -0.18) |
| **Systolic blood pressure (mmHg)** | 3,694 (3 RCTs) | RCT | Not serious | Not serious | Not serious | Not serious | ⊕⊕⊕⊕ High | MD -4.32 mmHg  (-5.61 to -3.03) |
| **Diastolic blood pressure (mmHg)** | 3,694 (3 RCTs) | RCT | Not serious | Not serious | Not serious | Serious (–1)⁴ | ⊕⊕⊕◯ Moderate | MD -1.00 mmHg  (-1.03 to -0.97) |
| **Total cholesterol  (% change)** | 4,255  (4 RCTs) | RCT | Not serious | Not serious | Not serious | Not serious | ⊕⊕⊕⊕ High | MD -6.29%  (-10.54 to -2.03) |
| **LDL-cholesterol  (% change)** | 4,255  (4 RCTs) | RCT | Not serious | Not serious | Not serious | Not serious | ⊕⊕⊕⊕ High | MD -4.14%  (-6.38 to -1.91) |
| **HDL-cholesterol  (% change)** | 4,255  (4 RCTs) | RCT | Not serious | Not serious | Not serious | Not serious | ⊕⊕⊕⊕ High | MD +3.31%  (1.66 to 4.97) |
| **VLDL-cholesterol (% change)** | 3,922  (3 RCTs) | RCT | Not serious | Not serious | Not serious | Not serious | ⊕⊕⊕⊕ High | MD -10.81%  (-14.10 to -7.51) |
| **Triglycerides  (% change)** | 4,255  (4 RCTs) | RCT | Not serious | Not serious | Not serious | Not serious | ⊕⊕⊕⊕ High | MD -10.90%  (-14.36 to -7.43) |
| **Nausea** | 4,287 (4 RCTs) | RCT | Not serious | Serious (–1)^5^ | Not serious | Serious (–1)^6^ | ⊕⊕◯◯ Low | RR 5.22  (2.60 to 10.48) |
| **Vomiting** | 4,287 (4 RCTs) | RCT | Not serious | Not serious | Not serious | Not serious | ⊕⊕⊕⊕ High | RR 3.24  (2.69 to 3.90) |
| **Diarrhea** | 4,287 (4 RCTs) | RCT | Not serious | Not serious | Not serious | Not serious | ⊕⊕⊕⊕ High | RR 2.35  (1.95 to 2.84) |
| **Constipation** | 4,287 (4 RCTs) | RCT | Not serious | Not serious | Not serious | Not serious | ⊕⊕⊕⊕ High | RR 2.86  (2.34 to 3.50) |
| **Dyspepsia** | 4,287 (4 RCTs) | RCT | Not serious | Not serious | Not serious | Not serious | ⊕⊕⊕⊕ High | RR 2.70  (2.08 to 3.50) |
| **Abdominal pain** | 3,954 (3 RCTs) | RCT | Not serious | Not serious | Not serious | Not serious | ⊕⊕⊕⊕ High | RR 1.90  (1.34 to 2.69) |
| **GERD** | 3,954 (3 RCTs) | RCT | Not serious | Not serious | Not serious | Not serious | ⊕⊕⊕⊕ High | RR 2.72  (2.72 to 4.15) |
| **Eructation** | 4,287 (4 RCTs) | RCT | Not serious | Not serious | Not serious | Not serious | ⊕⊕⊕⊕ High | RR 6.80  (3.76 to 12.29) |
| **Headache** | 3,954 (3 RCTs) | RCT | Not serious | Not serious | Not serious | Serious (–1)^7^ | ⊕⊕⊕◯ Moderate | RR 1.28  (0.99 to 1.60) |

¹ I² = 84%, largely explained by dose and one outlier (Rosenstock 2025), ² Moderate-high I² in higher-dose subgroups + some imputed SDs, ³ Very high I² ≥96% in several dose subgroups, ⁴ Confidence interval includes no effect, ^5^ I² = 54%, entirely explained by Wharton 2025 (leave-one-out → I² = 0%), ^6^ Very wide CI (spans from mild to very large increase), ^7^ CI includes no effect (p = 0.06).

**Figure S3: Subgroup Analysis by Orforglipron for Changes in Weight Reduction (%)**


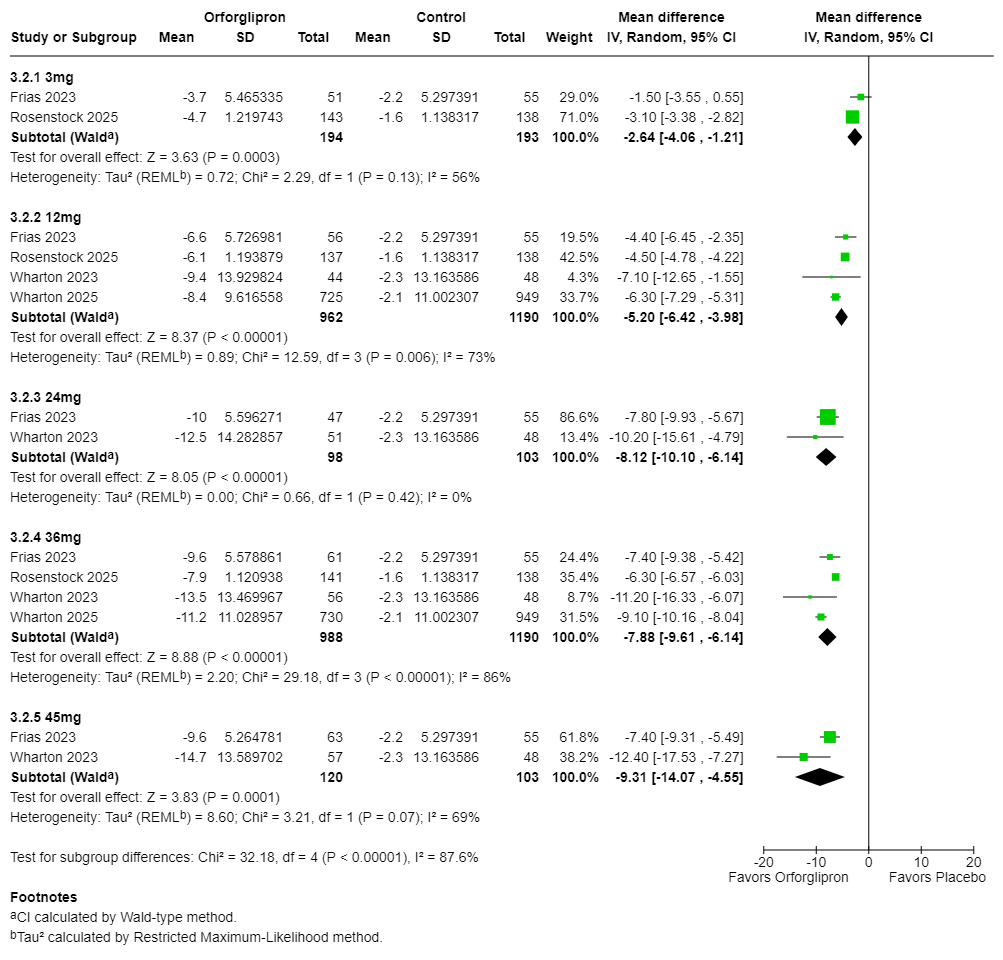


**Figure S3:** Forest plot of mean difference in percentage change in weight from baseline versus placebo, stratified by orforglipron daily dose. Random-effects model.

**Figure S4: Subgroup Analysis by Orforglipron Dose for reduction of in Waist Circumference (cm)**


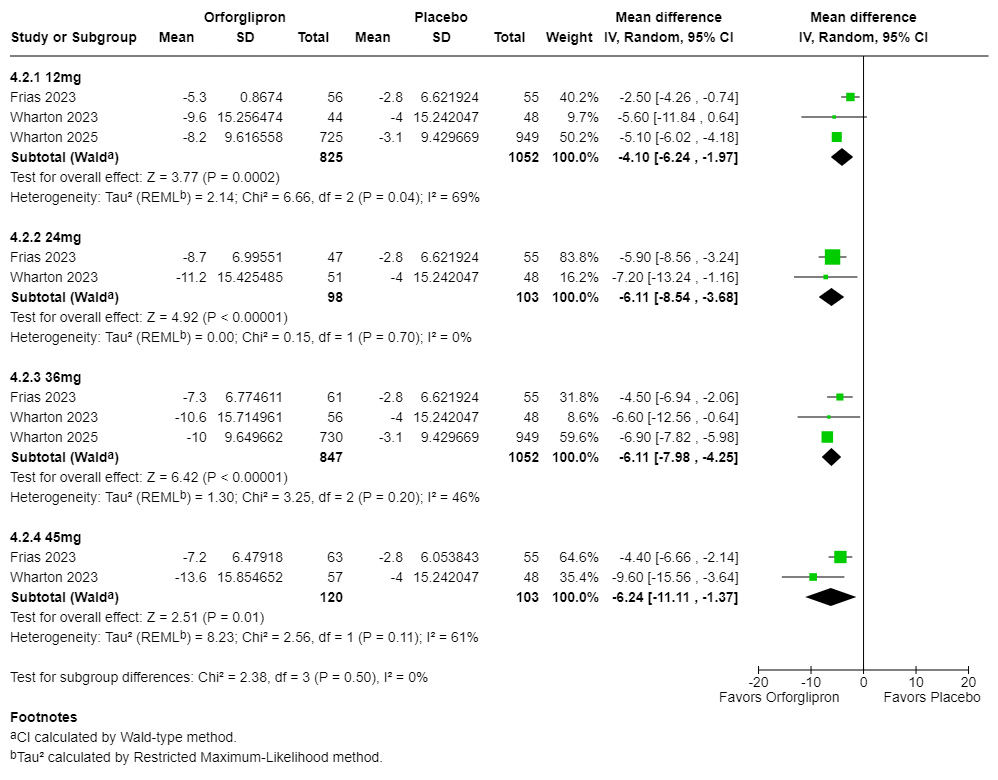


**Figure S4:** Forest plot of mean difference in percentage change in waist circumference from baseline versus placebo, stratified by orforglipron daily dose. Random-effects model.

**Figure S5: Subgroup Analysis by Orforglipron Dose for reduction of Systolic Blood Pressure (mmHg)**


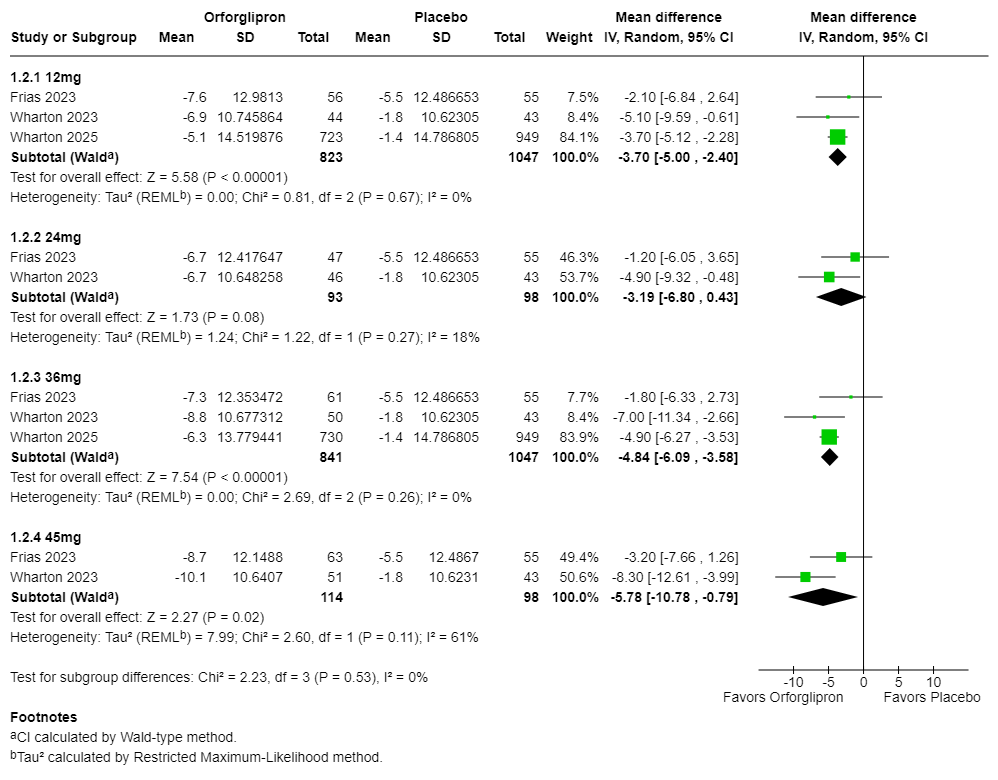


**Figure S5:** Forest plot of mean difference change in Systolic Blood Pressure (mmHg) from baseline versus placebo, stratified by orforglipron daily dose. Random-effects model.

**Figure S6: Pooled Effect of Orforglipron on Diastolic blood pressure**


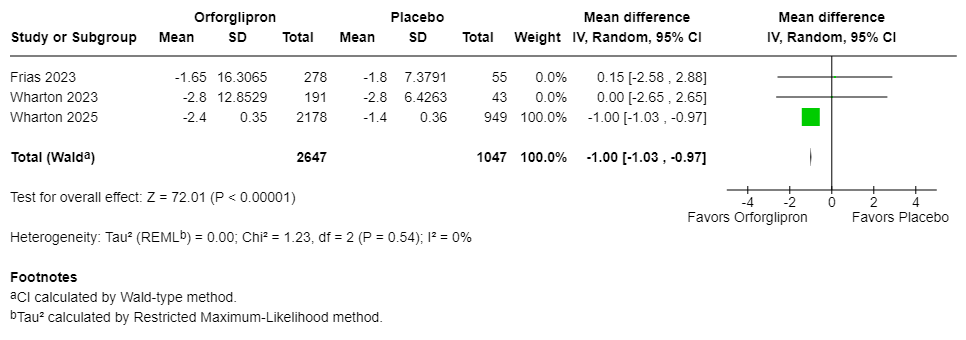


**Figure S6:** Forest plot of mean difference change in Diastolic Blood Pressure (mmHg) from baseline versus placebo, pooled analysis. Random-effects model.

**Figure S7: Subgroup Analysis by Orforglipron Dose for reduction of Diastolic Blood Pressure (mmHg)**


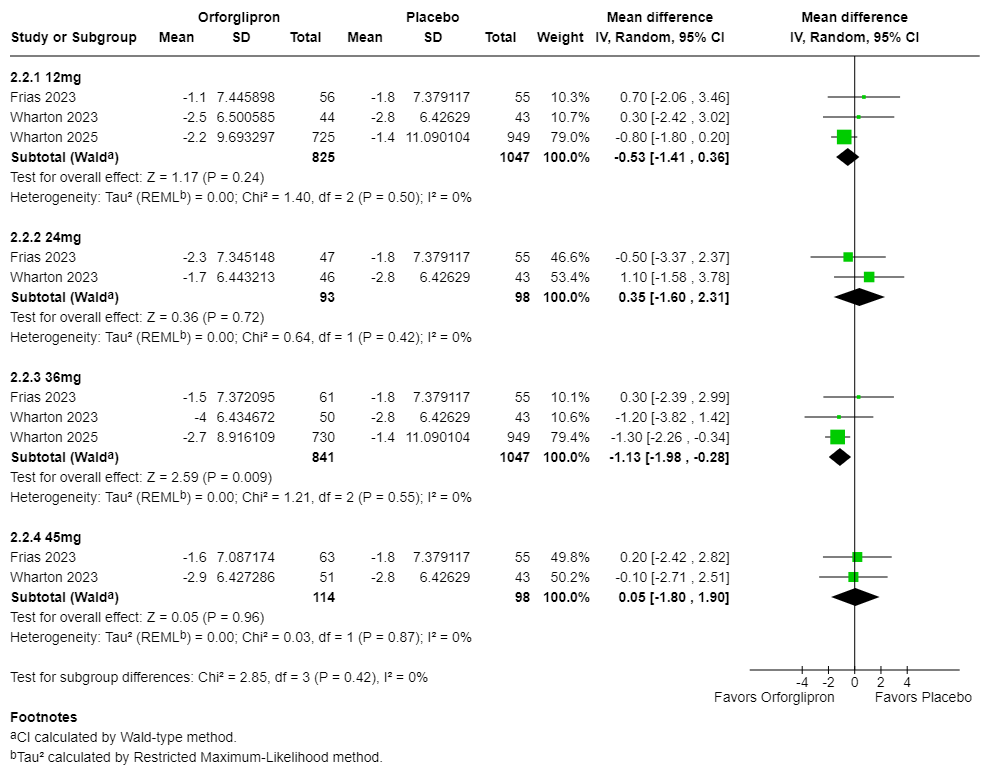


**Figure S7:** Forest plot of mean difference in percentage change in Diastolic Blood Pressure from baseline versus placebo, stratified by orforglipron daily dose. Random-effects model.

**Figure S8: Subgroup Analysis by Orforglipron Dose for Percentage Change in Total Cholesterol**


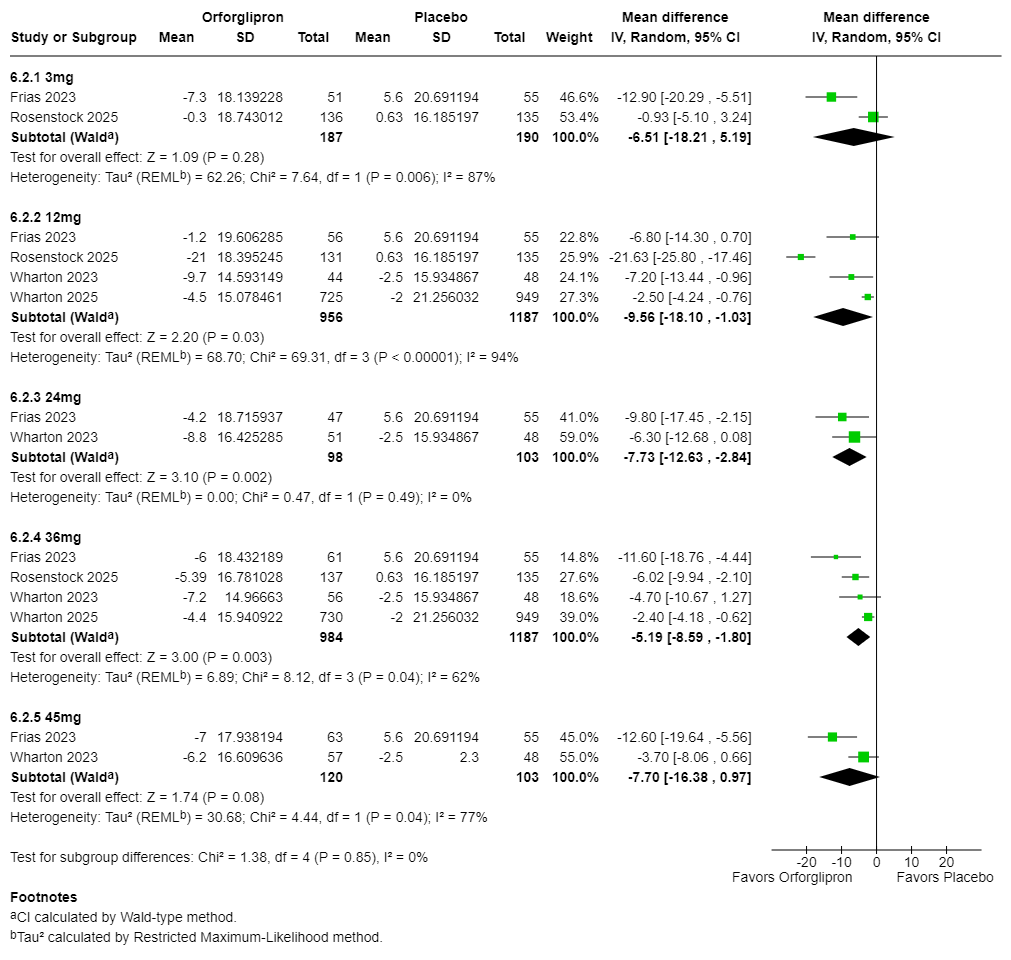


**Figure S8:** Forest plot of mean difference in percentage change in Total cholesterol from baseline versus placebo, stratified by orforglipron daily dose. Random-effects model.

**Figure S9: Subgroup Analysis by Orforglipron Dose for Percentage Change in HDL Cholesterol**


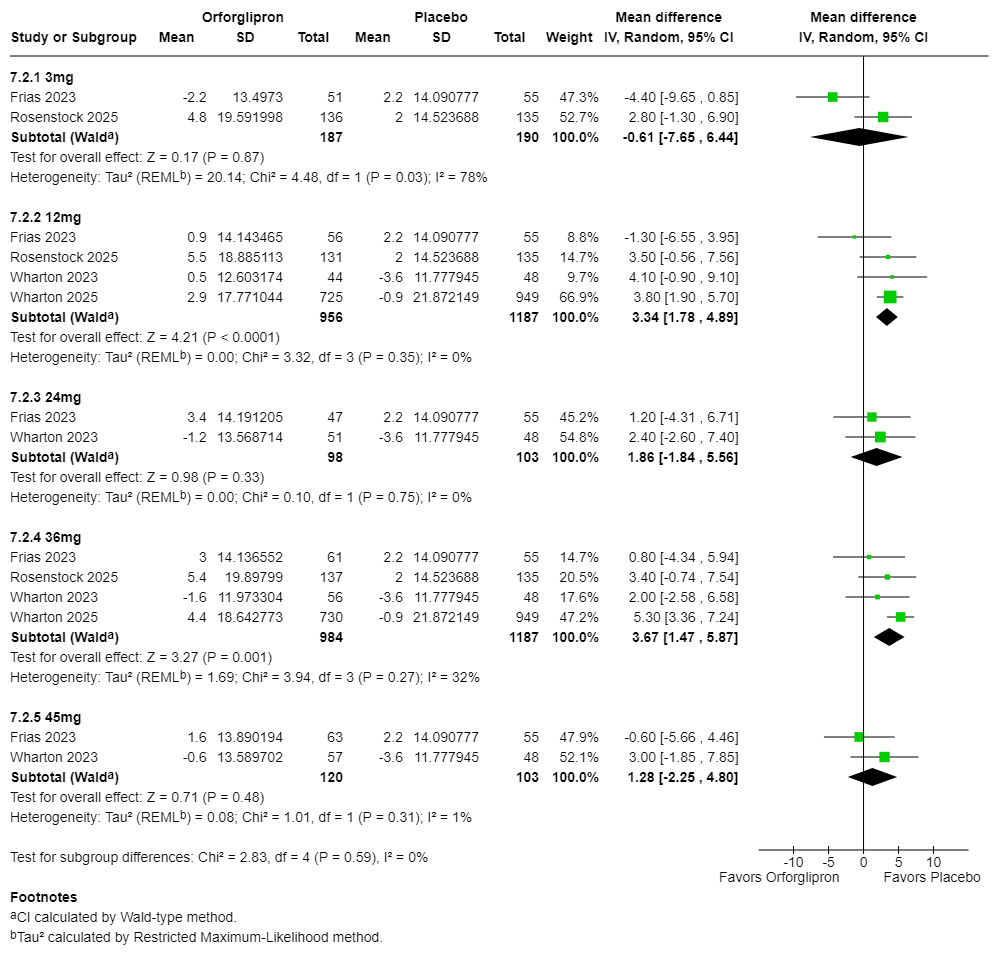


**Figure S9:** Forest plot of mean difference in percentage change in HDL cholesterol from baseline versus placebo, stratified by orforglipron daily dose. Random-effects model.

**Figure S10: Subgroup Analysis by Orforglipron Dose for Percentage Change in LDL Cholesterol**


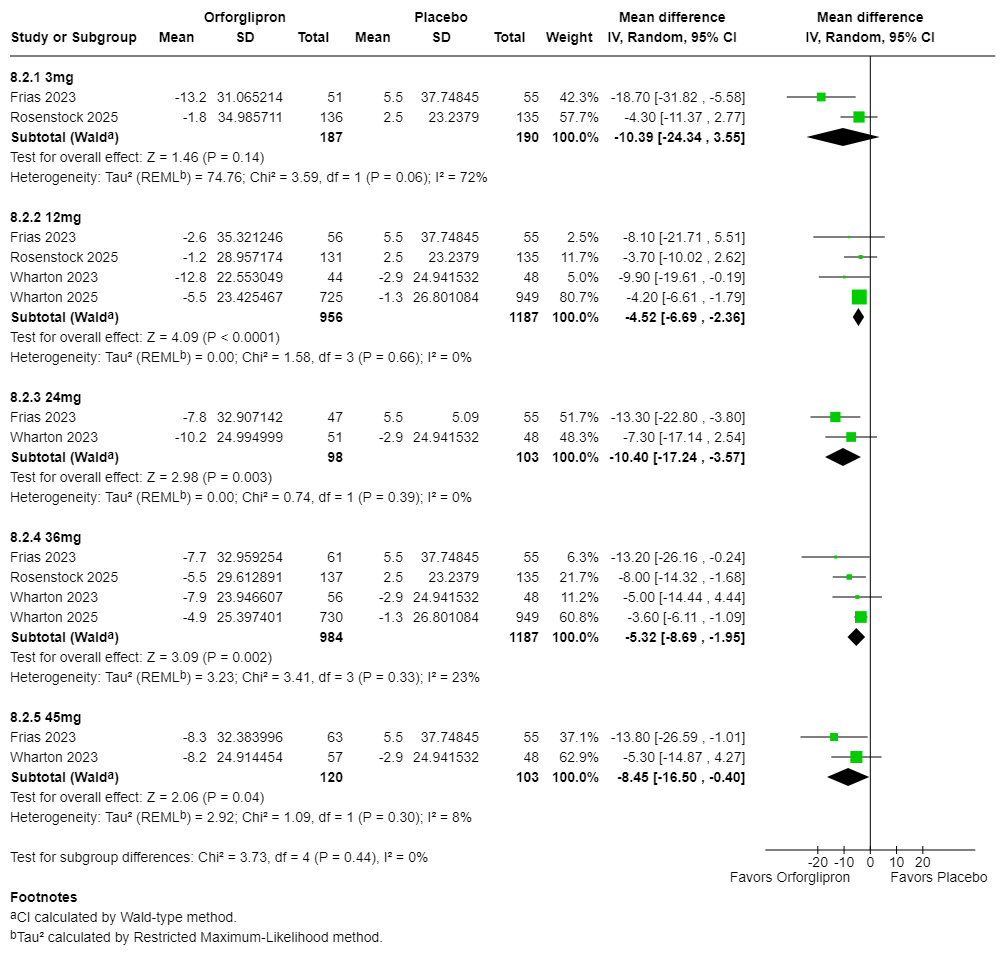


**Figure S10:** Forest plot of mean difference in percentage change in LDL cholesterol from baseline versus placebo, stratified by orforglipron daily dose. Random-effects model.

**Figure S11: Pooled Effect of Orforglipron on VLDL Cholesterol**


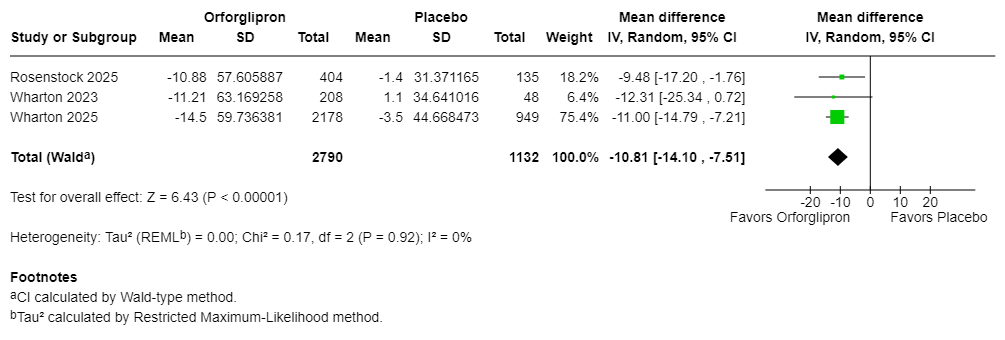


**Figure S11**: Forest plot of mean difference in percentage change in VLDL cholesterol from baseline with orforglipron versus placeb, pooled analysis. Random-effects model

###

###

**Figure S12: Subgroup Analysis by Orforglipron Dose for Percentage Change in VLDL Cholesterol**


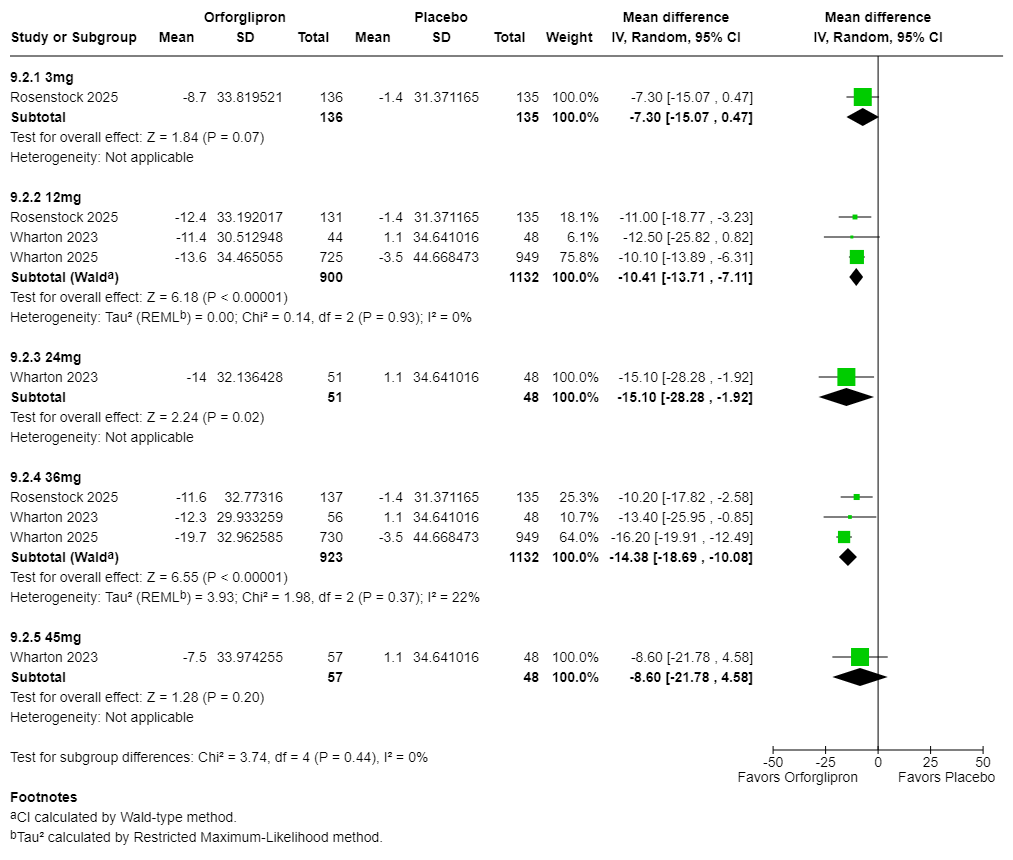


**Figure S12:** Forest plot of mean difference in percentage change in VLDL cholesterol from baseline versus placebo, stratified by orforglipron daily dose. Random-effects model.

**Figure S13: Pooled Effect of Orforglipron on Triglycerides**


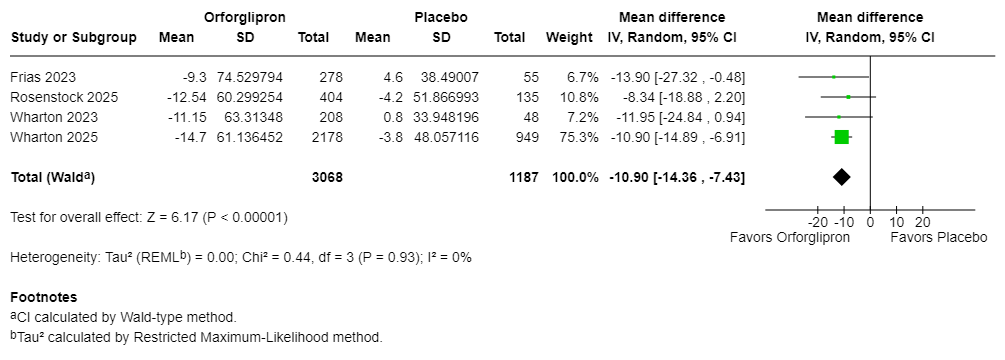


**Figure S13:** Forest plot of mean difference in percentage change in triglycerides from baseline with orforglipron (%) versus placebo across all doses, Pooled analysis, Random-effects model.

**Figure S14: Subgroup Analysis by Orforglipron Dose for Percentage Change in Triglycerides**

**Figure S14:** Forest plot of mean difference in percentage change in triglycerides from baseline versus placebo, sub-group analysis stratified by dosage of Orforglipron, Random-effects model.


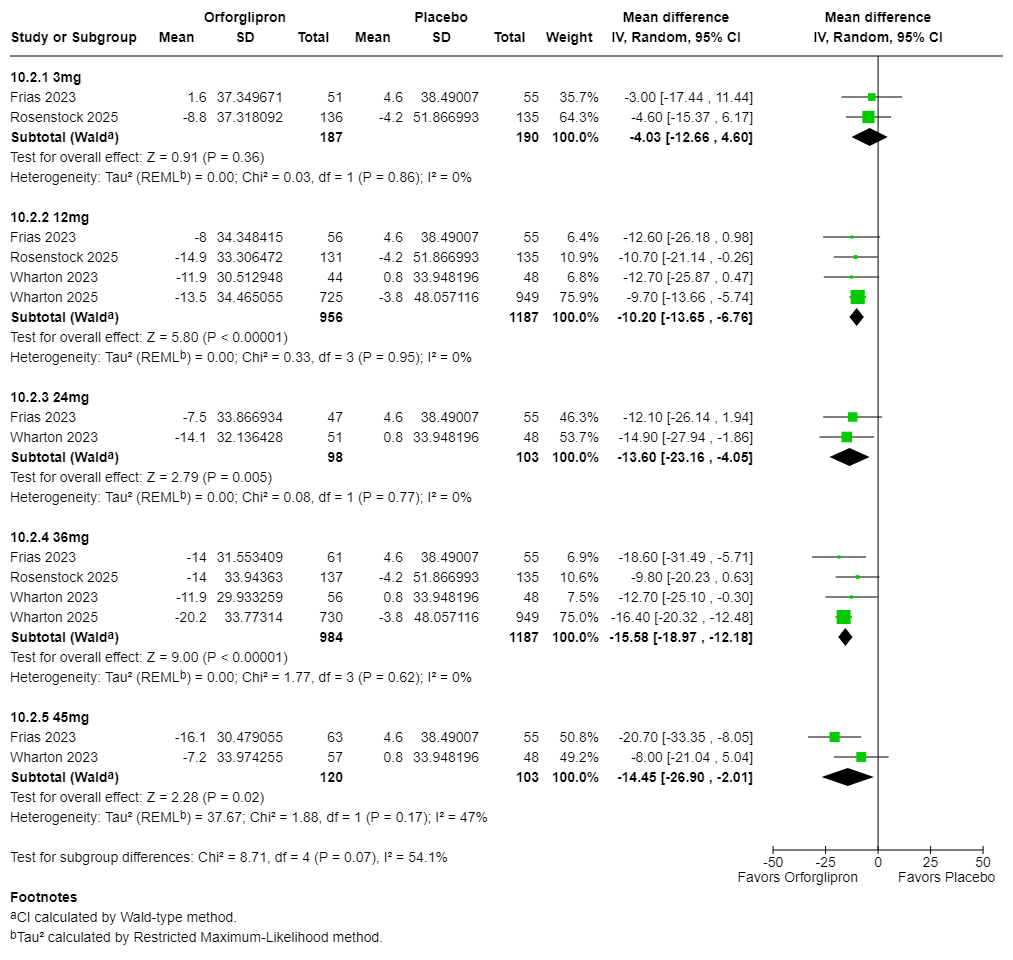


**Figure S15: Subgroup Analysis by Orforglipron Dose for Change in HbA1c (%)**


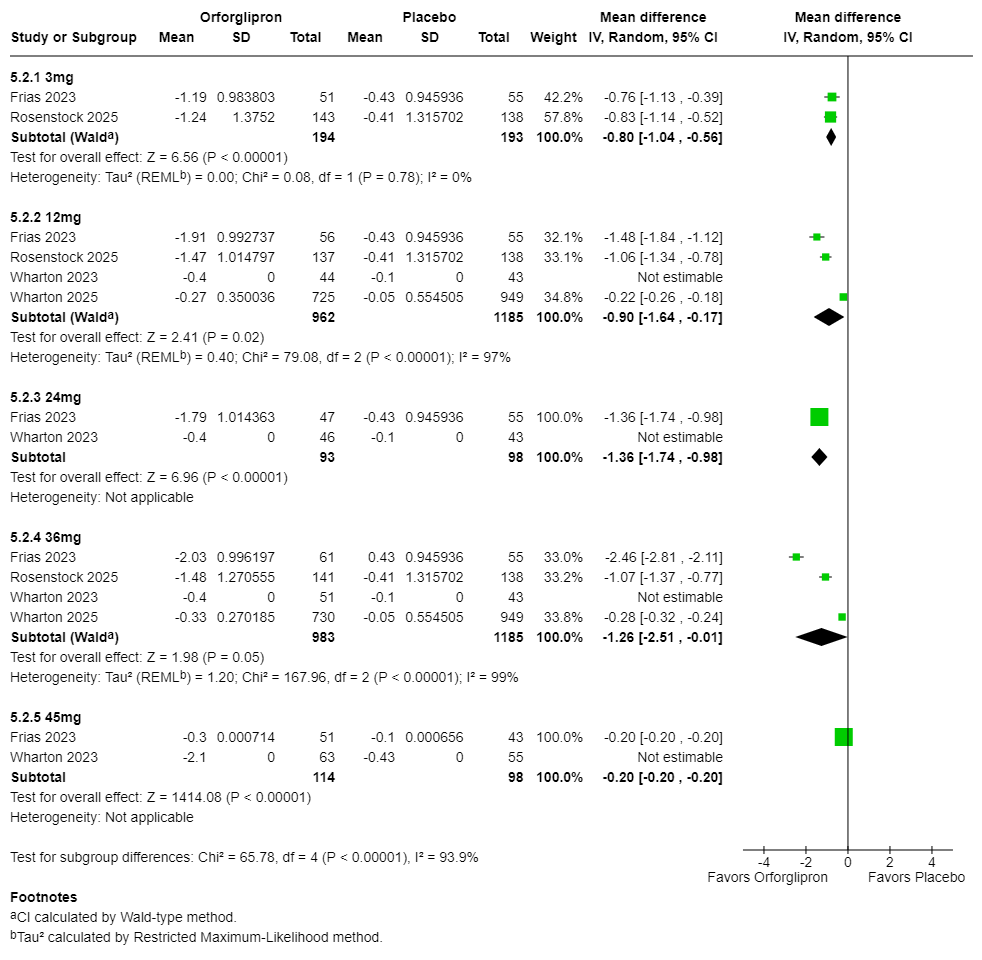


**Figure S15:** Forest plot of mean difference in HbA1c (%) from baseline versus placebo, sub-group analysis stratified by dosage of Orforglipron. Random-effects model.

**Figure S16: Treatment discontinuation due to any adverse event**


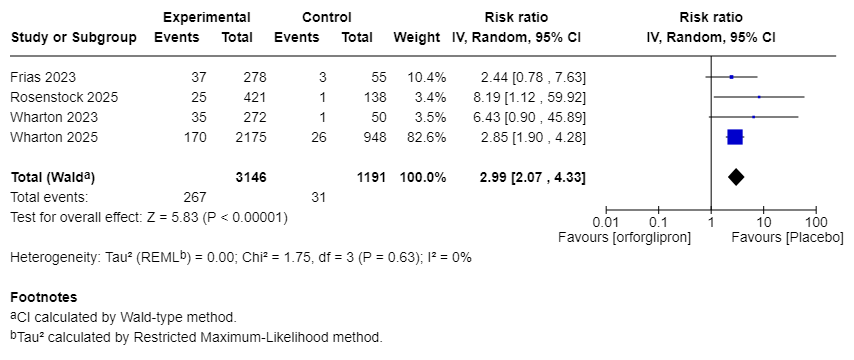


**Figure S16:** Forest plot of risk ratios for permanent treatment discontinuation due to adverse events (all causes) with Orforglipron versus placebo in four randomized controlled trials. Random-effects model. The diamond represents the pooled risk ratio and 95% CI.

**Table S17: Comparative cardiometabolic efficacy and tolerability of orforglipron versus established GLP-1 and GIP receptor agonists**

| **Agent** | **Route and highest evaluated dose** | **Body weight change (%)** | **HbA1c change (%)** | **Systolic blood pressure change (mmHg)** | **LDL-cholesterol change (%)** | **Triglycerides change  (%)** | **Discontinuation due to gastrointestinal adverse events (%)** |
| --- | --- | --- | --- | --- | --- | --- | --- |
| Orforglipron (present meta-analysis) | Oral once daily,  24–45 mg | -8.25% to -9.31% | -0.85% | -3.19 to -5.78 mmHg | -10.4 to -8.45 | -13.60 to -14.45 | 2.9–21% |
| Subcutaneous semaglutide^1^ | Subcutaneous weekly, 2.4 mg | -12.4% | -0.29% | -5.1 mmHg | -3% | -16% | 3.7% |
| Subcutaneous Liraglutide^2,3^ | Subcutaneous, 3.0 mg daily | -5.4% | -1.2% | -2.8 mmHg | -2.4% | -9.3% | 6% |
| Oral semaglutide^4,5^ | Oral once daily, 14 mg | -3.4% | -0.7% | -3 mmHg | -8% | -10% | 5.2% |
| Tirzepatide (dual GIP/GLP-1 RA)^6^ | Subcutaneous, 15 mg weekly | -12.0% | -0.3% | -5.8 mmHg | -2.1% | -33% | 5% |

Values represent placebo-adjusted mean changes from baseline at primary endpoint (26–72 weeks) derived from phase 3 programmes or large network meta-analyses. Ranges reflect results across the highest approved or evaluated doses in adults with type 2 diabetes or obesity without diabetes. Discontinuation rates reflect permanent treatment cessation attributed primarily to gastrointestinal adverse events. GLP-1 RA = glucagon-like peptide-1 receptor agonist; GIP = glucose-dependent insulinotropic polypeptide.

**References:**

1. Jastreboff AM, Aronne LJ, Ahmad NN, Wharton S, Connery L, Alves B, Kiyosue A, Zhang S, Liu B, Bunck MC, et al. Tirzepatide Once Weekly for the Treatment of Obesity. *N Engl J Med*. 2022;387:205–216.

2. Pi-Sunyer X, Astrup A, Fujioka K, Greenway F, Halpern A, Krempf M, Lau DCW, Le Roux CW, Violante Ortiz R, Jensen CB, et al. A Randomized, Controlled Trial of 3.0 mg of Liraglutide in Weight Management. *N Engl J Med*. 2015;373:11–22.

3. Le Roux CW, Astrup A, Fujioka K, Greenway F, Lau DCW, Van Gaal L, Ortiz RV, Wilding JPH, Skjøth TV, Manning LS, et al. 3 years of liraglutide versus placebo for type 2 diabetes risk reduction and weight management in individuals with prediabetes: a randomised, double-blind trial. *The Lancet*. 2017;389:1399–1409.

4. Husain M, Birkenfeld AL, Donsmark M, Dungan K, Eliaschewitz FG, Franco DR, Jeppesen OK, Lingvay I, Mosenzon O, Pedersen SD, et al. Oral Semaglutide and Cardiovascular Outcomes in Patients with Type 2 Diabetes. *N Engl J Med*. 2019;381:841–851.

5. Aroda VR, Rosenstock J, Terauchi Y, Altuntas Y, Lalic NM, Morales Villegas EC, Jeppesen OK, Christiansen E, Hertz CL, Haluzík M, et al. PIONEER 1: Randomized Clinical Trial of the Efficacy and Safety of Oral Semaglutide Monotherapy in Comparison With Placebo in Patients With Type 2 Diabetes. *Diabetes Care*. 2019;42:1724–1732.

6. Zhao L, Cheng Z, Lu Y, Liu M, Chen H, Zhang M, Wang R, Yuan Y, Li X. Tirzepatide for Weight Reduction in Chinese Adults With Obesity: The SURMOUNT-CN Randomized Clinical Trial. *JAMA*. 2024;332:551.
